# T1-nerve root neuroma presenting with apical mass and Horner's syndrome

**DOI:** 10.1186/1749-7221-2-7

**Published:** 2007-03-19

**Authors:** Roman Bošnjak, Urška Bačovnik, Simon Podnar, Mitja Benedičič

**Affiliations:** 1 Department of Neurosurgery, Division of Surgery, University Medical Center, Ljubljana, Slovenia; 2 Institute of Clinical Neurophysiology, Division of Neurology, University Medical Center, Ljubljana, Slovenia

## Abstract

**Background:**

The appearance of dumbbell neuroma of the first thoracic root is extremely rare. The extradural component of a T1-dumbbell neuroma may present as an apical mass. The diagnosis of hand weakness is complex and may be delayed in T1-neuroma because of absence of the palpable cervical mass. One-stage removal of a T1-root neuroma and its intrathoracic extension demanded an extended posterior midline approach in the sitting position.

**Case presentation:**

A 51-year old man had suffered a traumatic partial tendon rupture of his wrist flexor muscles 6 years ago. Since the incident he occasionally felt fullness and tenderness in the affected forearm with some tingling in his fingers bilaterally. During the last two years the hand weakness was continuous and hypotrophy of the medial flexor and intrinsic hand muscles had become apparent. Electrophysiological studies revealed an ulnar neuropathy in addition to mild median and radial nerve dysfunction, including a mild contralateral carpal tunnel syndrome. The diagnostic work-up for multiple mononeuropathy in the upper extremity was negative. Repeated electrophysiological studies revealed fibrillations in the C7 paravertebral muscles on the affected side. Chest x-ray revealed a large round apical mass on the affected side. A Horner's syndrome was noted at this point of diagnostic work-up. MRI of the cervical and thoracic spine revealed a dumbbell T1 neuroma enlarging the intervertebral foramen at T1-2 and a 5 cm large extradural tumor with extension into the apex of the ipsilateral lung. The patient underwent surgery in sitting position using a left dorsal midline approach. Although the T1 root could not be preserved, the patient's neurological condition was unchanged after the surgery.

**Conclusion:**

Extended posterior midline exposure described here using hemilaminectomy, unilateral facetectomy and costo-transversectomy is efficient and safe for one-stage removal of dumbbell tumors at the T1 level with a predominantly extraforaminal component in the apex of the lung extending up to 6–7 cm laterally. Horner's syndrome, if present and observed, may significantly narrow the differential diagnosis of hand weakness caused by T1-root tumors.

## Background

Neuromas (schwannomas and neurofibromas) are benign slowly growing peripheral nerve-sheath tumors originating from Schwann cells [1-3]. In the brachial plexus they account for 80% of primary tumors [1,3]. Schwannomas are composed entirely of Schwann cells, whilst neurofibromas contain Schwann cells, fibroblasts, perineurial cells, mast cells and axons in an extracellular matrix. Dumbbell neuromas with extradural components present a special entity of primary brachial plexus tumors and account for 15% of all cervical neuromas [4]. Their appearance in lower cervical roots is rare[4]. The extradural component of a T1-neuroma may present as an apical mass [5]. The diagnosis of T1 root neuromas may be particularly complex and delayed due to absence of a palpable cervical mass [1]. Most often, they mimic lesions of multiple nerves or nerve roots. However, in this tumor location, Horner's syndrome, if present and noticed, may significantly narrow the differential diagnosis of the hand weakness [5,6].

Three commonly used surgical approaches to the brachial plexus – supraclavicular, transaxillary and dorsal subscapular [5,7] enable relatively good exposure of the proximal brachial plexus, but do not allow access into the spinal canal and foramen as a single-stage microsurgical procedure.

We present a patient with a T1-root neuroma with significant lateral extension from the intervertebral foramen into the thoracic cavity. Apart from being a diagnostic challenge, tumors in this location also demanded a tailored single-stage surgical approach to the inferior proximal brachial plexus and spinal canal using an extended posterior midline approach in the sitting position.

## Case presentation

A 51-year-old right handed non-smoker experienced acute onset pain in his left forearm, following a traumatic episode 6 years previously, when a box fell onto his forearm. A partial rupture of the wrist flexor muscle tendons was diagnosed. Following this, he noticed ipsilateral hand weakness and tingling in his fingers a few months later. During the subsequent two years following his initial traumatic episode, his hand weakness worsened, particularly in the winter and subsided in the summer. However, during the last two years the weakness had become permanent. He experienced difficulty in buttoning his shirt and grasping a glass with his left hand. He also noticed wasting of the left hand muscles and complained of bilateral finger paraesthesia during the night, mainly on the right.

Neurological examination of the left upper extremity revealed mild hypotrophy of the ulnar flexors and intrinsic hand muscles (hypothenar and first dorsal interosseous). In addition, he had moderate weakness of wrist flexion, and finger abduction (4/5 MRC). Sensory testing revealed mild hypoaesthesia of the medial arm, forearm and the little and ring fingers. His reflexes were all preserved.

### Investigations

**Figure 1:**
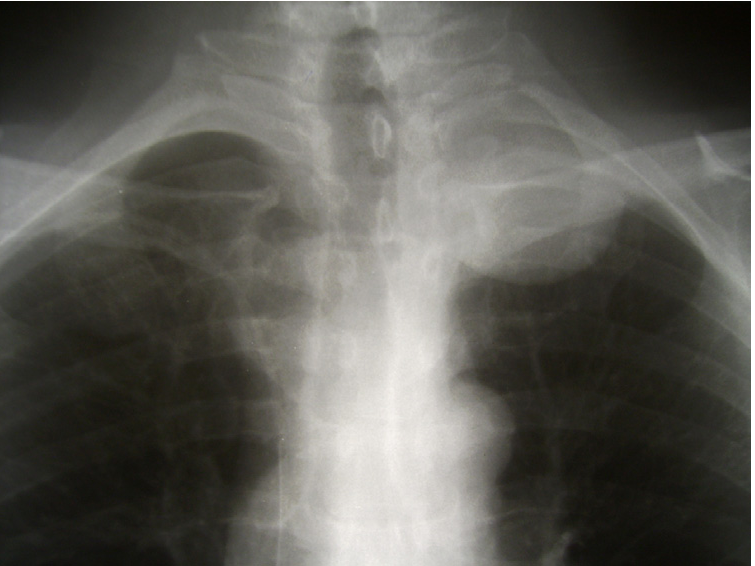
Pre-operative chest X-ray demonstrating a round shadow of approx. 5 cm in diameter in the left pulmonary apex.

**Figure 2:**
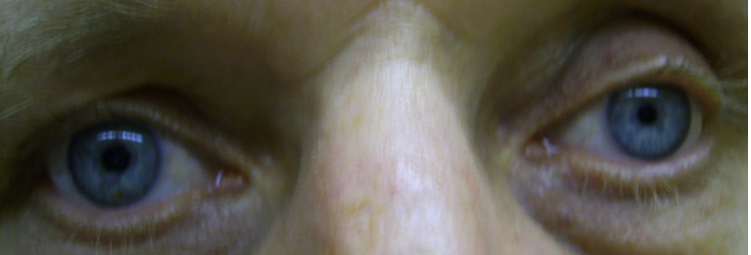
Left sided miosis due to T1 lesion – incomplete Horner's syndrome.

**Figure 3:**
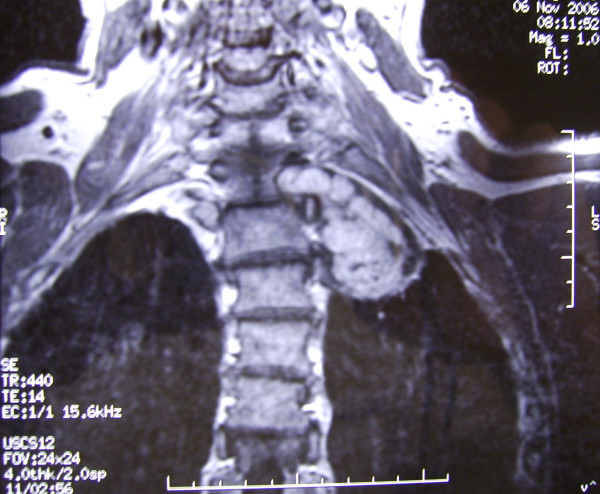
Coronal section of T1 weighted MRI demonstrating the left pulmonary apex tumor with extension into T1-2 intervertebral foramen.

**Figure 4:**
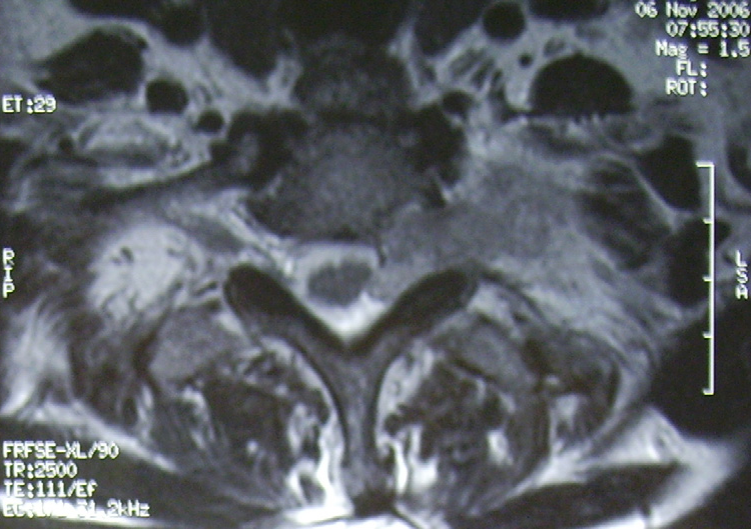
Transverse section of T1 weighted MRI demonstrating protrusion of the tumor from the left T1-2 intervertebral foramen to the spinal cord.

Electrophysiological examination demonstrated a reduction in amplitude of the median nerve M-wave (left: 1.2, right 10.1 mV), with no F-waves (detection from the abductor pollicis brevis muscle). Furthermore, reduced median nerve sensory conduction velocities were noted across the wrist on the right. Amplitudes of the left ulnar (4^th ^and 5^th ^fingers) and median (2^nd ^and 4^th ^fingers) sensory nerve action potentials were similar to the right. Concentric needle electromyography (EMG) revealed denervation activity in the flexor carpi radialis, the first dorsal interosseous muscles, and chronic reinnervation changes in the extensor indicis, flexor carpi radialis and abductor pollicis brevis muscles. Additional electrophysiological examinations revealed mild denervation in the left paravertebral muscles, and no nerve conduction or needle EMG abnormality in the lower limbs. Further diagnostic work-up was tailored to reveal the etiology of the upper extremity multiple mononeuropathy. B12, folic acid, TSH, lues, HIV, Hep-2, and boreliosis were all negative. Radiographs of the cervical spine revealed intervertebral hondrosis and dorsal osteophytes at C5-6 and C6-7. The chest x-ray revealed a round lesion in the apex of the left lung (Figure 1). A subtle left sided Horner's syndrome was noted afterwards (Figure 2). The CT scan of the thorax confirmed a left apical extrapulmonary tumor and enlargement of the T1-2 intervertebral foramen. MRI of the cervical and upper thoracic spine revealed some minor intraspinal protrusion of the foraminal tumor and delineated a 5 cm large solid extraforaminal tumor with intense, inhomogenous enhancement (Figures 3, 4). The sympathetic innervation of the skin in the face was normal as revealed by the starch – jodid test.

### Surgery

**Figure 5:**
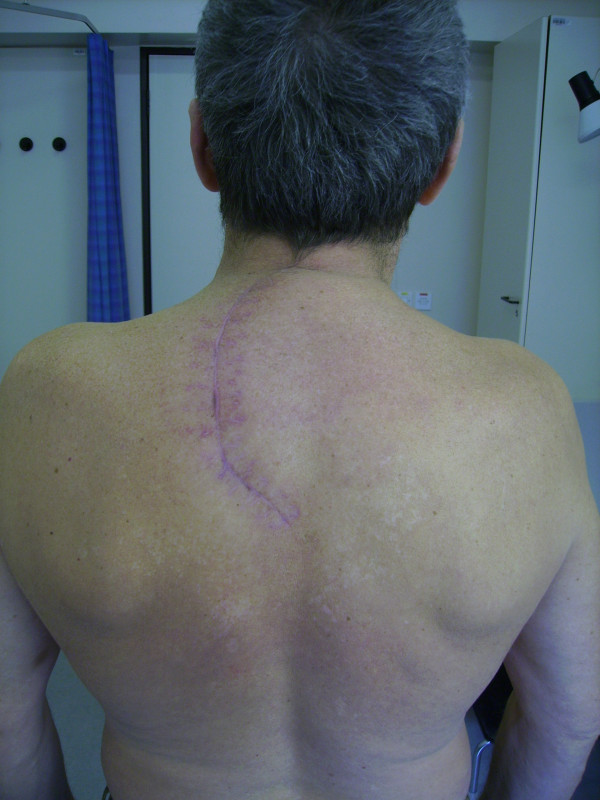
A single curvilinear paramedian incision in the sitting position of the patient allowed for posterior midline approach and dorsal subscapular approach under the same skin flap if necessary.

**Figure 6:**
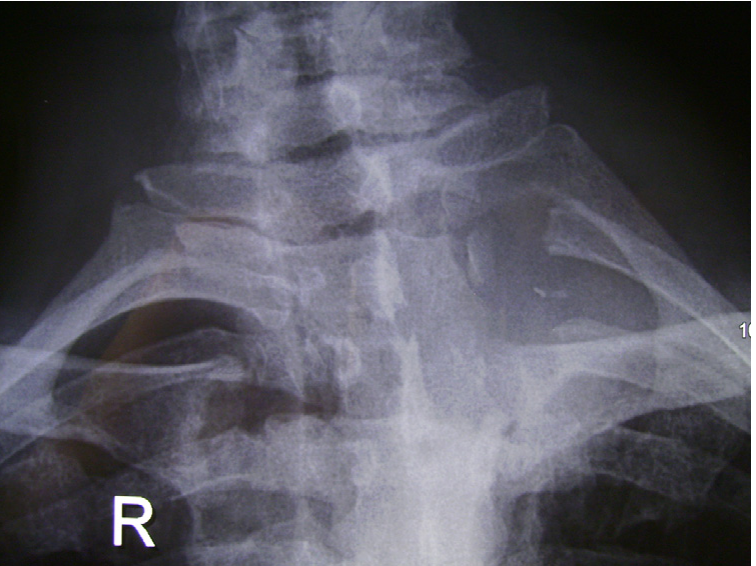
Post-operative chest X-ray demonstrating removal of the T2 and T3 transverse processes, and proximal parts of the second and third ribs.

**Figure 7:**
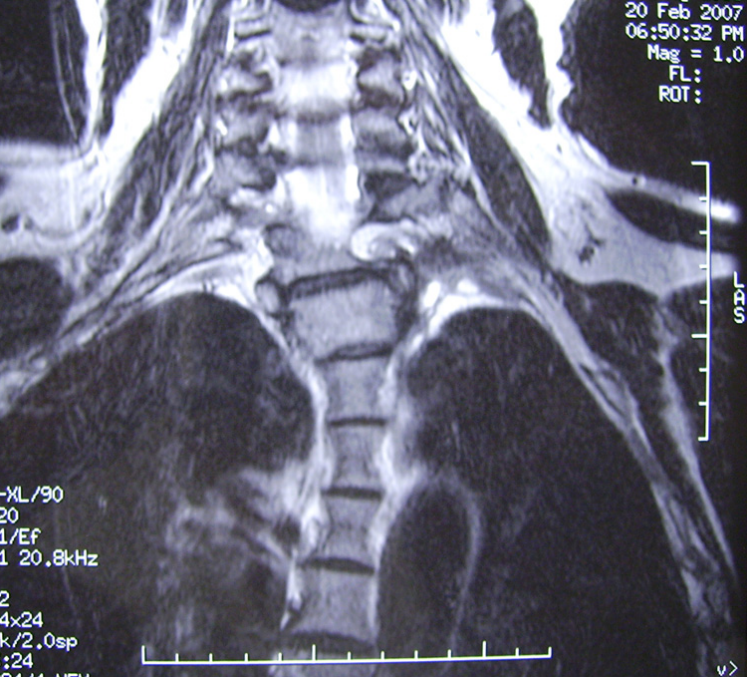
Coronal section of T1 weighted MRI demonstrating complete removal of the left-sided T1-neuroma from the T1-2 intervertebral foramen and from the pulmonary apex.

**Figure 8:**
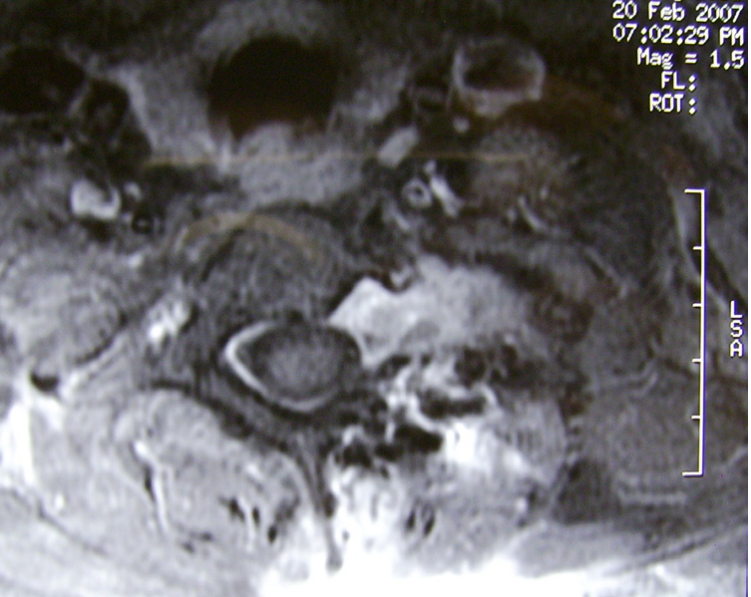
Transverse section of T2 weighted MRI demonstrating complete removal of the neuroma from the left T1-2 intervertebral foramen and the spinal canal.

The patient underwent surgery in the sitting position. A left-sided paravertebral curvilinear incision was made from C6 to T4, up to 5 cm lateral in its central part, and the skin flap was turned medially to expose the midline (Figure 5). The cervicothoracic fascia was incised on the left side just lateral to the spinous processes C7-T3. Paravertebral muscles were bluntly dissected away from spinous processes to expose the left hemilaminae of T1-3, facet joints T1-2 and T2-3, and the transverse processes T1-3. The left hemilaminae of T1 and T2 were removed as well as the left facet joint T1-2. The left transverse processes T2 and T3 and proximal parts of the ribs 2 and 3, up to the costo-transverse joints were drilled away (Figure 6). The T1 dural sleeve was enlarged and filled with the tumor in the distal tree-quarters of length, but the most proximal part of the dural sleeve was nearly normal in width. Under microscopic magnification a 2.5 cm long vertical incision into the left lateral dural sac was first performed to explore T1-rootlets intraspinally where entering into the T1-dural sleeve. A brown-yellowish looking tumor was found to protrude from the dural sleeve into the spinal canal and dislocate the rootlets peripherally, but did not reach the spinal cord. Then the dural sleeve was longitudinally incised and opened. The ventral and dorsal T1 rootlets were found free in the most proximal part of the dural sleeve, but after 4–5 mm they were completely lost in the tumor. Stimulation of the fascicles in the proximal dural sleeve revealed no motor response in the hand, and therefore, the rootlets were sacrificed at this point. Tumor was completely removed from the intervertebral foramen, the dural sleeve was circumferentially cut between the middle and proximal third of its length. It is sometimes very hard to close the dura water-tightly, but in our patient the closure was successful because the most proximal part of the dural sleeve was normal and preserved as a stump. This short proximal stump of the T1-dural sleeve was folded, sutured to the dural sac and glued. The dura closure was easier because the exploratory vertical incision of the dural sac and the longitudinal incision of the the dural sleeve were not joined and were separately closed by sutures. The tumor in the apex was first hollowed piece-meal and then removed from the parietal pleura. The last part of the tumor was found attached to the distal end of the T1 spinal nerve, just proximal to its union with the C8 spinal nerve forming the inferior trunk of brachial plexus, and divided. Immediately posteriorly, the subclavian artery was observed. Complete extracapsular removal of the tumor was possible (Figures 7, 8).

### Postoperative course

The postoperative course was uneventful. Immediately after surgery the patient demonstrated identical hand and finger function as preoperatively (see Additional file 1). Several days later at discharge he reported subjective improvement in the opposition of the thumb and index finger on the left side. Furthermore, the mild tingling in the medial side of the arm and ulnar side of the forearm had disappeared. The sensory deficit in the upper extermity remained unchanged. No additional neurological deficits were noted.

### Pathology

The tumor specimen revealed densely packed spindle-shaped cells on microscopic examination. Cells were diffusely S100 imuno-marker positive. Some cells were also positive to EMA. NF marked some rare axons. Ki-67 was 2%. Due to the focal appearance of whorl-like tumors cells and their EMA imunopositivity (as seen in meningiomas) the pathological diagnosis of an atypical schwannoma was made. These peculiar neuropathological features in schwannoma are more often seen as a part of neurofibromatosis but the patient didn't fulfill the clinical criteria for neurofibromatosis. However, genetic analysis was not performed.

## Discussion

A complex morphology and unique functional anatomy make a diagnostic work-up of brachial plexus lesions challenging even to the experienced. Significant inter-individual variations can further mask the clinical picture and make precise localization these lesions even more challenging [8]. In patients with neurogenic tumors, the clinical picture evolves slowly not only due to the slow tumor growth, but also due to collateral reinnervation [1]. Some symptoms and signs may also be intermittent or position-related because of local mass effect. In the absence of a palpable mass in the supraclavicular fossa the diagnosis is often delayed [1]. The first clue aiding diagnosis of brachial plexus lesions is involvement of multiple peripheral nerves or multiple roots. These can be better characterized by electromyography, which will also demonstrate the chronic reinnervation phenomenon.

In our patient, the initial clinical findings were not sufficient to explain the ulnar motor neuropathy, in addition to the sensory loss in the forearm and arm. The situation was further masked by night paraesthesia in the fingers bilaterally. Electrodiagnostic studies confirmed denervation changes in the muscles innervated by the median nerve (the flexor carpi radialis), and ulnar nerves (the first dorsasl interosseous). Furthermore, chronic reinnervation changes were also found in muscles (the extensor indicis) innervated by the radial nerve. These findings made a proximal lesion more likely, and this was further supported by denervation changes in the cervical paravertebral muscles. However, the particularly intriguing feature was the patients' history of symptoms abating during the summer, and reappearing during the winter. This broadened the differential diagnosis, to include the possibility of autoimmune neuropathy. This possibility was further supported by normal EMG findings in biceps brachii, triceps brachii and pectoralis major muscles. Nerve conduction studies demonstrated mild median neuropathy at the wrist compatible with carpal tunnel syndrome. Symmetric sensory nerve action potentials detected in the fingers were compatible with a preganglionic location or with nerve conduction block. The diagnosis was unexpectedly aided by the routine chest x-ray. After a repeated thorough clinical examination, it was noted that the patient had a miosis of the left pupil, suggestive of Horner's syndrome. Our patient nicely demonstrates that a Horner's syndrome, if present and observed, along with a long history and slowly progression of hand weakness due to involvement of multiple nerves or roots indicates possibility of a C8 or T1 spinal root tumor. In such patients even a simple chest x-ray may provide crucial diagnostic information.

What makes tumor in our patient exceptional is not only its location in the intraspinal, foraminal and extraforaminal compartments, but even more its extension into the thoracic cavity, projecting dorsally behind the first 3 ribs and laterally to the costo-transverse joints.

At the start of surgery, identification and preservation of the functioning spinal root fascicles should be performed. The functionality of the fascicles can be checked by direct electrical stimulation, and recording the response from appropriate muscles.

The midline approach to the intraspinal and foraminal part of the tumor enables preservation of these fascicles by early proximal identification of the subarachnoid rootlets in the dural sac and sleeve, and then following them by intrafascicular dissection into the foraminal component of the tumor [4,9,10]. Three commonly used approaches to the brachial plexus allow relatively good exposure of the proximal brachial plexus [5]. However, they do not allow access into the spinal canal and foramen as a single-stage procedure and are therefore combined with posterior midline approach in one-stage or two-stage surgery. A supraclavicular approach in the supine position enables easy identification of proximal roots, trunks and vessels, but the T1 tumor is located underneath all these structures [11]. Lot and George reported removal of C8 dumbbell neuromas as a caudal limit of their anterolateral approach [4]. A transaxillary approach in the lateral decubitus position enables early visualization of the inferior trunk, but a thoracotomy and retraction of the parietal pleura are necessary to access the caudal part of the tumor. Similarly, a posterior subscapular [7] approach also provides extensive inferior brachial plexus exposure, which can be carried proximally to the foramen, but posterior resection of the first rib is also required.

We decided to put our patient in the sitting position and performed a single curvilinear paramedian incision (Figure 5). Such incision allowed for posterior midline approach and dorsal subscapular approach under the same skin flap. Because it was initially not clear, whether we would be able to access the most lateral part of the tumor with midline approach, a lateral intermuscular approach as done in dorsal subscapular approach was planned as a secondary option. Many authors claim that additional, more lateral approaches are necessary in the same stage or as a second-stage procedure to remove tumor components that extend more than 4–5 cm from the lateral dural margin, which is probably true for cervical dumbbell tumors [4,10]. However, the extended posterior midline exposure described here provided access to the most lateral (up to 7 cm from the lateral dural margin) aspects of the tumor. It can be seen in Figure 1 that half of the 5 cm large apical tumor was located lateral to the costo-transverse joints. However, tumor debulking was essential for such a laterally localized lesion.

Technical advancements have introduced other possibilities for removing these apical mass dumbbell neuromas of the T1 root in a combined approach using transthoracic endoscopic surgery [5,12]. However, this approach does not allow for nerve root preservation. A similar approach to T2 root neuromas with apical extension has also been reported [13]. Standard midline exposure includes unilateral hemilaminectomy of the adjacent laminas and unilateral facetectomy for full exposure of the intraspinal and intraforaminal tumor [10], but complementary video-assisted thoracoscopic surgery may avoid unilateral facetectomy in certain neuromas. In dumbbell neuromas without intradural extension, Han and Dickman [14] suggested truncation of the tumor at the foramen followed by removal of the head and neck of the rib and some portion of the rostral pedicle of the lower vertebral body to follow the tumor into the enlarged foramen and divide the root there. Avulsion injury to the spinal cord and roots was not reported. On the contrary, Barranchea et al. divided roots in the dural sac first, then removed the intradural and preganglionic intraforaminal tumor, and pushed the remaining tumor with the distal stump of the nerve into the chest cavity via the enlarged foramen [12]. Sparing of the facet joint in the combined microsurgical-thoracoscopic approach is not justified in T1-dumbell neuromas, where the root preservation should be always attempted.

We did not perform spinal fusion in our patient, because we feel similar to other authors that complete unilateral facetectomy in combination with hemilaminectomy does not bear a significant risk of spinal instability [4,10].

Most studies confirmed that for complete tumor removal the affected root needs to be sacrificed, with relatively low risk of severe permanent postoperative injury. McCormick [10] reported significant radicular motor deficits in 1 out of 12 patients, and subjective transient radicular complaints in 2 of 12 (17%) patients. Kim *et al*. [15] noted mild, partial deficits in 7 of 31 (23%) patients, compared to Schultheiss and Gullotta [16] who reported mild, transient motor deficits in 1 of 10 patients. Celli [9] and Seppala et al. [17] reported similar results. The main mechanism of compensation for these root lesions is collateral axonal reinnervation. The inter-individual variations in pattern of poly-radicular innervation of muscles and skin can further compensate for gradual axonal loss of the affected root. The frequency of root transection is higher in neurofibromas (77%) than in schwannomas (31.8%) due to histological pattern seen in neurofibromas [4,9,17].

## Conclusion

The extended posterior midline exposure described here using hemilaminectomy, unilateral facetectomy and costo-transversectomy is useful for a one-stage microsurgical removal of dumbbell tumors in the T1 level with a predominant extraforaminal component extending to the apex of the lungs. It provides contiguous exposure of the intraspinal, foraminal and extraforaminal region, which extends up to 7 cm from the lateral dural margin. The key of our technique is piecemeal tumor debulking similar to the way in which intracranial tumors are resected. No second skin incision, wound extension, or repositioning are necessary with this approach. However, two-staged surgery may be beneficial in elderly or patients with carotid artery stenosis, cardiomyopathy, coronary heart disease, history of pulmonary embolism or trombembolisms, etc. where the sitting position is contraindicated.

## Competing interests

The author(s) declare that they have no competing interests.

## Authors' contributions

RB performed the surgery, concepted and drafted the manuscript together with UB and MB, who also clinically examined the patient and made the appropriate literature review. SP performed the conduction and electromyographic studies and helped drafting the manuscript with critical remarks.

All authors read and approved the final manuscript.

## Supplementary Material

Additional file 1 Postoperatively the patient demonstrated identical hand and finger function as preoperatively. This short movie shows hand and finger function after surgery. Click here for file
